# Assessing cognitive processes with diffusion model analyses*:* a tutorial based on *fast-dm-30*

**DOI:** 10.3389/fpsyg.2015.00336

**Published:** 2015-03-27

**Authors:** Andreas Voss, Jochen Voss, Veronika Lerche

**Affiliations:** ^1^Psychologisches Institut, Ruprecht-Karls-Universität HeidelbergHeidelberg, Germany; ^2^School of Mathematics, University of LeedsLeeds, UK

**Keywords:** *fast-dm*, diffusion model, parameter estimation, response time distribution

## Abstract

Diffusion models can be used to infer cognitive processes involved in fast binary decision tasks. The model assumes that information is accumulated continuously until one of two thresholds is hit. In the analysis, response time distributions from numerous trials of the decision task are used to estimate a set of parameters mapping distinct cognitive processes. In recent years, diffusion model analyses have become more and more popular in different fields of psychology. This increased popularity is based on the recent development of several software solutions for the parameter estimation. Although these programs make the application of the model relatively easy, there is a shortage of knowledge about different steps of a state-of-the-art diffusion model study. In this paper, we give a concise tutorial on diffusion modeling, and we present *fast-dm-30*, a thoroughly revised and extended version of the *fast-dm* software (Voss and Voss, 2007) for diffusion model data analysis. The most important improvement of the *fast-dm* version is the possibility to choose between different optimization criteria (i.e., Maximum Likelihood, Chi-Square, and Kolmogorov-Smirnov), which differ in applicability for different data sets.

Six years ago, we published *fast-dm-26* (Voss and Voss, 2007). Since then, applications of diffusion models have thrived in different domains of psychology (Voss et al., 2013a): Although diffusion models are still far from being a standard method in the cognitive sciences, they are now successfully applied by many different researchers addressing a wide variety of research questions. Different aims of the application of diffusion models can roughly be grouped into three groups.

A first type of diffusion model studies is interested in the development of cognitive models, and—specifically—in demonstrating that the diffusion model adequately describes the ongoing cognitive processes (e.g., Ratcliff, 1978; Ratcliff et al., 2004). For such studies, the key objective is demonstration of a good model fit, because a satisfactory model fit supports the assumption that actual cognitive processes are similar to the processes presumed by the model.

Secondly, diffusion models can be used to test predictions from psychological theories (e.g., Voss et al., 2013b). For such studies the validity of the diffusion model for the applied task should be undisputed. The application of the diffusion model aims at getting valid measures for specific cognitive processes, which then are entered into further statistical analyses as dependent variables. With this technique it becomes possible to explain *why* response latencies are shorter in one condition compared to another condition. As detailed below, in the diffusion model framework, faster responses can be based on (1) fast information processing, (2) low response thresholds, or (3) fast response execution.

Recently, a third—related—type of question has been addressed by diffusion model accounts: These are studies that use diffusion models as a diagnostic tool (e.g., Schmiedek et al., 2007; White et al., 2010). Diffusion models provide valid criteria for cognitive processes which, subsequently, can be related to other measures. For example, speed of information processing might be a proxy for intelligence (Schmiedek et al., 2007; Ratcliff et al., 2008, 2010) and a low response threshold might predict impulsive behavior.

In parallel to these applications of the model, many important theoretical and methodological advances helped to promote diffusion modeling in psychology. Most important, the development of user-friendly software solutions cleared the path for this kind of analyses. Available solutions comprise the *EZ*-method (Wagenmakers et al., 2007, 2008b; Grasman et al., 2009), *DMAT* for Matlab (Vandekerckhove and Tuerlinckx, 2007, 2008), *fast-dm* (version 29: Voss and Voss, 2007, 2008), and most recently, two Bayesian implementations for (hierarchical) diffusion models (Vandekerckhove et al., 2011; Wiecki et al., 2013). All these programs have special advantages, because they differ in (a) the mathematical methods used for parameter estimation (e.g., optimization criteria), (b) their flexibility to adapt to different complex data (e.g., experiments with multiple conditions), and (c) the usability and handling of the programs.

With this paper we want to introduce a new and extended version of *fast-dm* (*fast-dm-30*). The new developments regard the following points:

1. The new version allows the user to choose between different optimization criteria (Kolmogorov-Smirnov, Chi-Square, and Maximum Likelihood). This allows optimizing parameter estimation for different data, because optimization criteria differ in robustness and efficiency depending on characteristics of data.
2. A new parameter measuring so-called response-execution biases has been implemented (Voss et al., 2010). This parameter allows for the non-decisional component to differ between the two possible responses.
3. The code was optimized and minor bugs have been removed, including problems of using command-line options on Windows systems.
4. The tools to simulate data (*construct-samples*), and to calculate predicted CDFs and density functions (*plot-cdf* and *plot-density*) have been improved and are now better documented.

In the following we will provide a short introduction to diffusion model analysis followed by a discussion of advantages and disadvantages of different optimization criteria. Then, we give a step-by-step tutorial how to run a diffusion model project. The paper concludes with a description of the handling of *fast-dm-30* and its accompanying tools.

## The basics: a short introduction to diffusion modeling

Diffusion models are a formal model of decision making, that is, they provide a mathematical framework to understand decisional processes. They belong to the continuous sampling models (Ratcliff and Smith, 2004): These models assume that information is continuously sampled during a decision phase until evidence is sufficiently clear. As soon as one of two thresholds is reached, a response is initiated. The information sampling is described by a Wiener Diffusion Process which is characterized by a constant systematic drift (*v*) and Gaussian noise. The drift determines the average slope of the diffusion process and can be interpreted as the speed of information uptake. The standard deviation of the random noise (diffusion constant) is a scaling parameter in diffusion model analyses: It has to be fixed to a specific value that defines the scale for all other diffusion model parameters^1^. *Fast-dm* uses a diffusion constant of *s* = 1, while other researchers prefer to use *s* = 0.1. To make solutions comparable, it is essential to transform estimates for drift (*v*), threshold separation (*a*), starting point (*z*), and the so-called intertrial variability of drift and starting point (*s*_*v*_ and *s*_*z*_) by the following equation:

(1) $$p_{new}\;=\;\frac{s_{new}}{s_{old}}p_{old},$$

where *p*_*new*_ and *p*_*old*_ are the transformed and the original estimates, and *s*_*new*_ and *s*_*old*_ are the diffusion constants.

A second characteristic of standard diffusion models is the assumption that the diffusion process runs in a corridor between two thresholds, and it is terminated when one of them is hit. These thresholds represent two alternative outcomes of the decision process; depending on which threshold is hit, different responses are executed. By convention the lower threshold is positioned at 0 on the decision dimension and the upper threshold at *a*. Thus, *a* gives the amount of information that separates both possible decisional outcomes. Larger threshold separations lead—on average—to longer durations of the decision process. At the same time, an increasing distance between thresholds renders it more unlikely that random influences drive the process to the threshold opposite of the drift; that is, decision errors become rarer.

Sometimes one decisional outcome might be preferred over the other. To reach the preferred decision less information might be needed than for the non-preferred decision. Such a bias is often denoted in psychology as *response bias* (e.g., in Signal Detection Theory, Green and Swets, 1966) to emphasize that this kind of bias is independent of the quality of information processing (or sensitivity). However, we prefer here the term *decisional bias* because this bias is also unrelated to processes of response *execution*. In diffusion modeling, such a decisional bias is mapped on the starting point (*z*), which is positioned between 0 and *a* on the decision dimension. The closer the starting point is positioned to one threshold, the less information is needed to decide for the associated option. The new version of *fast-dm* uses the relative starting point (*z*_*r*_) for input and output. The relative starting point is defined as *z*_*r*_ = *z*/*a* (range: 0–1; *z*_*r*_ = 0.5 indicates unbiased decisions).

Obviously, the diffusion process as described so far cannot account for the total chain of information processing. Depending on the task, there will be additional processes of preparing for a task and encoding of stimuli that take place before a decision phase starts. After the decision is reached, motor processes have to be executed. The diffusion model sums the duration of all extra-decisional processes into one additional parameter, denoted as non-decisional component *t*_0_ (or sometimes *T*_*er*_, for Time of Encoding and Response) measuring the total duration of those processes. Total response time is assumed to be the sum of the duration of the decisional processes (mapped the diffusion process) and the non-decisional processes (*t*_0_).

The new version of *fast-dm* allows for different durations of motor processes for both outcomes (Voss et al., 2010). This might be relevant if one response is pre-activated (e.g., by response priming, Voss et al., 2013b), or if it is executed more (or less) frequently (e.g., in rare target search). In the implementation of the two execution times in *fast-dm*, a common *t*_0_ parameter is used, giving the average duration of non-decisional processes, and a difference parameter *d*, giving the difference of duration of non-decisional processes for the responses connected to the lower vs. upper threshold. These parameters can be re-transformed into separate *t*_0_ parameters with

(2a) $$t_{0}(upper\;threshold)\;=\;t_{0}-0.5\;\cdot \;d$$

(2b) $$t_{0}(lower\;threshold)\;=\;t_{0}+0.5\;\cdot \;d.$$

Most diffusion model analyses also take into account trial-to-trial fluctuations in cognitive components. For example, it is implausible to assume that participants' attention is equal throughout an experiment of several hundreds of trials; thus speed of information uptake (i.e., the drift) might differ slightly from trial to trial. Fluctuations in drift may also arise from different stimuli that are employed in different trials of an experiment. Similar points can be made for the inter-trial variability of starting point and of duration of non-decisional processes. For these reasons, most applications of the diffusion model allow for inter-trial variability of the drift (*v*), starting point (*z*), and non-decision constant (*t*_0_). Specifically, the actual drift is assumed to follow a normal distribution with mean *v* and standard deviation *s*_*v*_. Starting point and non-decisional constant follow uniform distributions with mean *z* and width *s*_z_, and mean *t*_0_ and width *s*_*t*0_, respectively. As for the starting point, *fast-dm-30* uses a relative measure for inter-trial-variability of staring points, with *s*_*zr*_ = *s*_*z*_/*a*.

The complete diffusion model as described above decomposes the decision process into 8 parameters (Table 1). Of course, models need not to include all of these parameters. Sometimes it might be better to make models more parsimonious by fixing parameters to given values. This regards specifically the starting point that can be fixed to *z*_*r*_ = 0.5 when no decision bias is expected (especially, when responses coded as false vs. correct), the response-time difference *d* that should be fixed to *d* = 0 when there is no reason to expect differences in speed of response execution, and the inter-trial variability parameters, that can be fixed to *s*_*v*_ = *s*_*zr*_ = *s*_*t*0_ = 0 when trial numbers are too small to allow for a robust estimation of these parameters.

**Table 1:** **Parameters of the Diffusion Model, typical ranges of values, and cognitive interpretation**.

**Parameter**	**Fast-dm**	**Typical range**	**Interpretation**
Drift	*v*	−4 to +4	average speed of information uptake
Threshold separation	*a*	0.6 to 2	response caution
Starting point	*z*_*r*_	0.4 to 0.6	decision bias
Non-decisional constant	*t*_0_	0.2 to 1.0	duration of non-decisional processes
Difference in non-decisional constant	*d*	−0.1 to +0.1	response preparation/response inhibition
Intertrial variability of drift	*s*_*v*_	0 to 1	differences in stimulus properties or fluctuations in attention
Intertrial variability of starting point	*s*_*zr*_	0.0 to 0.5	differences in expectations
Intertrial variability of non-decisional constant	*s*_*t*0_	0 to 1	differences in speed of response execution

Ranges for parameters refer to a diffusion constant of s = 1.

On the other hand, diffusion models often comprise more than the 8 parameters described above: Typically, different values for one parameter are estimated for different types of stimuli or different experimental conditions.

## How to estimate parameters: a comparison of different optimization criteria

A diffusion model analysis is based on the multi-dimensional search for an optimal set of estimates for all free parameters, so that there is a close fit between predicted and observed response time distributions. Since the RT distribution is split into two parts—for responses connected to the upper and lower threshold—the probability of responses (e.g., the error rate) is implicitly contained by the RT distributions. For the parameter search an optimization criterion has to be defined that quantifies the match between predicted and observed distributions. The most important improvement of *fast-dm-30* is that the user can now choose between three different optimization criteria: In addition to the Kolmogorov-Smirnov (KS) criterion that was used exclusively in *fast-dm*-29, we now implemented the commonly used Chi-Square (CS) approach and a Maximum Likelihood (ML) based algorithm. Because all algorithms have specific advantages, we will consider each of them below. Further information on the technical implementation of the algorithms is given in the section on technical details.

### Maximum likelihood (ML)

ML algorithms are highly efficient and are broadly applied to optimization problems for different models. In the case of diffusion models, the natural logarithms of density values (*g*)—calculated from predicted RT-distributions—are summed over all trials *i* (with response time *RT*_*i*_ and response *k*_*i*_):

(3) $$LL\;=\;\sum ln\left(g(RT_{i},k_{i})\right)$$

To make the algorithm more robust, a minimum value for density of *g* = 10^−6^ is used in *fast-dm*, that is, *g* is set to 10^−6^, when the predicted density is smaller than this value. The parameter search procedure then maximizes the resulting log-likelihood value. Because the ML procedure is highly efficient, it is especially useful in the case of small trial numbers. With the ML method parameters of parsimonious models may be estimated accurately from only 50 trials or less (Lerche et al., submitted). However, ML methods are especially sensitive to (fast) outliers. Even if only one (or very few) responses are added at the lower edge of the RT distribution, the accuracy of results will be derogated dramatically.

An additional advantage of the ML approach is that it allows the calculation of information criteria to compare different models. For example, the Bayesian Information Criteria (BIC) could be used here (Fific et al., 2010):

(4) BIC = −​2 ln(LL) − P · ln(M),

where *P* is the number of free parameters and *M* in the number of observations (i.e., trials).

### Chi-square (CS)

Th CS criterion has been frequently used in diffusion model approaches (Ratcliff and Tuerlinckx, 2002). The main advantages are the very fast calculation and its robustness against outliers. The computed CS value is based on the comparison of the number of observed and predicted responses in so-called bins of the RT-distributions. The borders of these bins are defined by convention by the 0.1, 0.3, 0.5, 0.7, and 0.9 quantiles of the empirical response time distributions, separately for the upper and lower threshold^2^. Thus, the optimization criterion is calculated across the 2 × 6 bins as

(5) $$CS\;=\;\sum \frac{{\left(o_{i}-p_{i}\right)}^{2}}{p_{i}},$$

with *o*_*i*_ and *p*_*i*_ being the observed and predicted, respectively, number of responses in bin *i*. The parameter search minimizes the CS value. If more experimental conditions are fitted simultaneously, CS values are added over conditions as well. Next to the advantages of fast calculation and its robustness, the CS approach comes with the benefit that the CS value can be taken as a test statistic for model fit. The degrees of freedom are then given by

(6) df = K(N−1)−P,

with *K* conditions of an experiment, *N* bins per condition (*N* = 2 · 6 = 12), and *P* free diffusion model parameters (White et al., 2010). A significant CS value indicates substantial misfit of the diffusion model. However, with large trial numbers, significant deviations are to be expected and other strategies of model tests might be preferable (Voss et al., 2013a).

Generally, CS based parameter estimations are only feasible for medium to large trial numbers (minimum 200 trials). It is especially problematic if empirical response distributions are small at one of the thresholds (e.g., less than 12 trials). In this case, the borders of bins are defined very unreliably. Unfortunately, this is often the case in diffusion model applications, where typically very easy tasks are used (e.g., lexical decision) and few errors occur. If in one experimental condition one response is given in less than 12 trials, *fast-dm-30* ignores these responses for the calculation of the CS value.

### Kolmogorov-smirnov

Previous versions of *fast-dm* only implemented the KS criterion (Voss and Voss, 2007). We originally opted for this approach because its characteristics can be seen as a compromise between ML and CS based methods: On the one hand, the KS method is efficient, because it is not based on binning responses but utilizes the complete distribution; on the other hand, the KS criterion is not as sensitive to outliers as is the ML criterion (Lerche et al., submitted).

The KS criterion is defined as the maximum absolute vertical distance between the empirical and the predicted cumulative density functions (CDF) of the response time distributions. Over *n* responses of an experiment, it can be computed as

(7) $$KS\;=\;{\text{max}}_{i\;=\;1\ldots n}\left|\text{eCDF}({\text{RT}}_{i})\text{-pCDF}({\text{RT}}_{i})\right|\text{​},$$

where *RT*_*i*_ is the response latency in trial *i*, and *eCDF* and *pCDF* are the empirical and predicted CDFs, respectively. In diffusion modeling there are always two empirical distributions to be compared with their predicted counterparts (i.e., the distributions linked to the two responses). In *fast-dm* this problem is solved by combining both distributions into one. This is achieved by multiplying all RTs from responses linked to the lower threshold with −1 (Voss et al., 2004; Voss and Voss, 2007). *Fast-dm* transforms KS-values in associated *p*-values (with *df* = *numberofresponses*), which are than maximized (Voss and Voss, 2007). In case of multiple experimental conditions, the product of all *p*-values from the different conditions is maximized.

Simulations from our lab (Lerche et al., submitted) show that—for uncontaminated data—the KS method tends to be slightly less efficient compared to the ML method but reveals notably more accurate results compared to the CS approach. For contaminated data, KS performs best in most cases.

## Some technical details

### The calculation of cumulative density functions (CDF)

The optimization routines based on the KS or CS statistics require the calculation of predicted CDFs. For the basic diffusion model (without inter-trial variabilities) the CDF for decision time *t* for responses at the upper threshold can be calculated as the solution of the following partial differential equation (PDE; see Voss and Voss, 2008):

(8a) $$\frac{\partial }{\partial t}F_{+}\left(t,z\right)\;=\;\frac{1}{2}\frac{\partial^{2}}{\partial z^{2}}F_{+}\left(t,z\right)+v\frac{\partial }{\partial z}F_{+}\left(t,z\right)$$

with boundary conditions

(8b) $$F_{+}\left(t,0\right)\;=\;0,\;F_{+}\left(t,a\right)\;=\;1,\text{ for all }t>0$$

and initial condition

(8c) $$F_{+}\left(0,z\right)=\{\begin{array}{cc} 0 & \;if\;0\leq z<a\\ 1 & if\;z=a \end{array}$$

It is possible to derive an explicit solution to this PDE that allows the direct calculation of the CDF (Ratcliff, 1978; Blurton et al., 2012). However, a numerical solution of the PDE introduced by Voss and Voss (2008) proved to be much faster while yielding the same accuracy, especially if inter-trial variability of starting point and non-decisional component are included in calculations. The PDE is solved numerically using a finite difference scheme, by discretizing the ranges of the starting point *z* and decision time *t* (see Press et al., 1992, Chap. 19, for an introduction to numerical solutions of PDEs). The accuracy of the solution depends on discretization steps sizes for *z* and *t*. In *fast-dm* a “precision” parameter allows to control step sizes used in the calculation of CDFs (see below).

### The calculation of density functions

For the ML approach, density functions have to be calculated. For the basic diffusion model (without inter-trial variabilities) there are two different representations of the density *g*_+_ for the first-passage time *t* of a diffusion process with starting point *z* and threshold separation *a* (Van Zandt et al., 2000; Voss et al., 2004; Navarro and Fuss, 2009):

(9a) $$\begin{array}{l} g_{+}\left(t,z,a,v\right)\;=\;\frac{exp\left[\left(a-z\right)v-0.5v^{2}t\right]}{\sqrt{2\pi t^{3}}}\\ \sum_{n=-\infty }^{\infty }exp\left(-\frac{{\left[\left(1+2n\right)a-z\right]}^{2}}{2t}\right)\;\cdot \;\left[\left(1+2n\right)a-z\right] \end{array}$$

and

(9b) $$\begin{array}{l} \;g_{+}\left(t,z,a,v\right)\;=\;\frac{\pi }{a^{2}}\exp\left[(a-z)v\right]\\ \sum_{n\;=\;1}^{\infty }n\;\cdot \;\sin\left(\frac{\pi (a-z)n}{a}\right)exp\left[-\text{​}0.5\left(v^{2}+\frac{\pi^{2}n^{2}}{a^{2}}t\right)\right]. \end{array}$$

Navarro and Fuss (2009) show that Equation (9a) converges quickly for small *t* and Equation (9b) converges quickly for large *t*. In *fast-dm-30*, we implemented this finding and calculate densities always with the equation that converges faster. The numbers of terms used to approximate the infinite series are chosen to keep a maximum error bound of 1e-6 (Navarro and Fuss, 2009).

The value *t* in Equations (9a) and (9b) is the decision time. The non-decision parameter *t*_0_ has to be subtracted from all empirical response times, before the densities are computed (*t* = RT-*t*_0_). The density of the distribution at the lower threshold (*g*_−_) can be easily obtained by replacing *v* with *-v* and *z* with *a-z*, respectively. To include inter-trial variabilities, *g_+_* has to be integrated over *v*, *z*, and *t*_0_.

(10) $$g^{\prime }(t,z,a,v,s_{v})\;=\;\int_{-\infty }^{\infty }g_{+}\left(t,z,a,v^{\prime }\right)\;\cdot \;\frac{1}{\sqrt{2\pi s_{v}^{2}}}e^{-\frac{{\left(v^{\prime }-v\right)}^{2}}{2s_{v}^{2}}}dv^{\prime }$$

(11) $$g^{\prime \prime }\left(t,z,a,v,s_{v},s_{z}\right)=\int_{z-0.5s_{z}}^{z+0.5s_{z}}\frac{g^{\prime }(t,z^{\prime },a,v,s_{v})}{s_{z}}dz^{\prime }$$

(12) $$g^{\prime \prime \prime }\left(t,z,a,v,s_{v},s_{z},s_{t0}\right)=\int_{t-0.5s_{t0}}^{t+0.5s_{t0}}\frac{g^{\prime \prime }(t^{\prime },z,a,v,s_{v},s_{z})}{s_{t0}}dt^{\prime }$$

The integral of Equation (10) can be solved analytically. Equations (11) and (12) are computed numerically in *fast-dm*; the discretization step size is controlled again by the precision settings (minimum number of steps is four). Thus, the precision settings take influence on the results (and calculation time) for the ML method only if inter-trial variability of *z* and/or *t*_0_ is greater than 0.

### Optimization routine

The optimization procedure is based on a multidimensional search for the optimal set of parameters that maximizes *p*(KS) or minimizes CS or -LL. For this procedure, we use an implementation of the SIMPLEX downhill algorithm (Nelder and Mead, 1965). This method is based on a simplex that comprises of *n* + 1 vectors of parameter values when *n* parameters are optimized. For the starting simplex, we use results from the *EZ*-method (Wagenmakers et al., 2007) for the first vector (with *z*_*r*_ = 0.5, and *s*_*v*_ = *s*_*z*_ = *s*_*t*0_ = 0), and variations where values for one parameter are increased by a small amount for the remaining vectors.

In our implementation of the simplex, we use two criteria simultaneously. Firstly, we penalize theoretically impossible parameter constellations (e.g., *z_*r*_* < 0, *z_*r*_* > 1, *a* < 0, etc.). For these cases, the optimization criteria cannot be calculated; solutions with penalty are always assumed to fit worse than any solution without penalty. The second criterion is the optimization criterion (*p*(KS), CS, or -LL). This second criterion is only used when no penalty is assigned to a solution. In case of KS, the corresponding *p*-value is minimized to allow the optimization of multiple experimental conditions.

Because the simplex algorithm is known to be unreliable in case of multidimensional search, we repeat the simplex search three times with different starting points and consecutively stricter stopping criteria.

## Planning, running, and interpreting diffusion model-analyses: a step-by-step guide

The following sections describe some important steps in a typical diffusion model analysis and provide some help on crucial choices that have to be made. An excellent general introduction in cognitive modeling is provided by Heathcote et al. (in press). Specific advices on fitting parameters to the related ballistic-accumulator model can be found in other tutorials (Donkin et al., 2009a, 2011).

### Step 1: choosing an experimental paradigm

If a study aims at a general investigation of cognitive processes (e.g., cognitive aging or practice effects) it is often to choose between different paradigms (i.e., experimental tasks) for a study. If this is the case, paradigms should be selected that have already been validated for diffusion model analyses. Such well-tested paradigms comprise—for example—recognition memory tasks (e.g., Ratcliff, 1978; Spaniol et al., 2006), numerosity or color-judgment tasks (e.g., Ratcliff, 2002; Voss et al., 2004), and lexical decision tasks (e.g., Ratcliff et al., 2004; Wagenmakers et al., 2008a).

Sometimes, however, it may be the aim of a project to investigate whether a specific (new) paradigm is apt for diffusion modeling. If this paradigm has not yet been validated for a diffusion model analysis before, it should be verified first that all theoretical prerequisites and assumptions of the model are met. We will explicate these assumptions below. Secondly, it needs to be shown empirically that model fit is satisfactorily (see Step 6), and finally, an empirical validation of model parameters is essential (Voss et al., 2013a). For example, in such a validation study it can be tested whether face-valid manipulation map on single parameters as expected (see Voss et al., 2004, for an example of an empirical validation).

Theoretical prerequisites of diffusion models are often neglected or addressed only implicitly. In the following, we give a short overview of basic assumptions (Voss et al., 2013a): Firstly, diffusion models assume a *continuous sampling of information*. This makes the model more suitable for tasks using stimuli containing conflicting information. A prototypical example is a field of pixels with two different colors. Here, it can be argued that color information is continuously sampled. In recognition tasks, not the stimuli itself are ambiguous; rather the familiarity (or the absence of familiarity) can be assumed to cumulate until a response is made.

Secondly, diffusion models require typically *binary decision* tasks. Optimally, diffusion model tasks should comprise two response keys that are linked in the analyses to the upper vs. lower threshold. It is also possible to recode responses as correct (upper threshold) vs. incorrect (lower threshold). However, this mapping requires some attention: (a) One needs to be sure that drift rates do not differ between stimulus types; (b) there should be no decision bias, and the relative starting point has to be fixed to 0.5; and (c) it has to be considered that results might be less robust in case of low error numbers (because then the distribution of responses at the lower threshold is absent or small). If these requirements are not met, the linear ballistic accumulator model should be preferred because it allows mapping data with multiple responses (Donkin et al., 2009b).

A third prerequisite refers to the assumption of *constancy of parameter values over time*. The Wiener diffusion model as described in this paper assumes that drift and threshold separation is constant over the time of a decision (and independent on the accumulated amount of evidence). The assumption of constant threshold separation might be violated when sparse information is present decision times are long. In this case, there shifts in criterion are highly plausible. However, the direction of such shifts remains rather unclear: It could be argued that participants will set more liberal criteria when they notice that they do not reach the conservative criteria after several seconds. On the other hand, it is possible that threshold separation is increased to avoid errors when the decision is really difficult. The assumption of constant drift could be violated when a stimulus changes over time (e.g., a hidden stimulus is continuously unmasked), or when it is removed from screen before a decision is reached (the drift might be stronger while the stimulus is present and weaker when it is only remembered).

Changes of drift rate over time might also occur in interference tasks like the stoop task or the flanker task, when distracting information has to be inhibited. The inhibition of irrelevant information might take some time, which results in an increase of drift rate during the decision phase.

A fourth assumption regards the required components of a task. The diffusion model is apt only for relatively simple *single-stage decisions*. More complex tasks that are composed of different steps (or insights) might again challenge the assumptions of continuous information sampling and constant drift.

### Step 2: how many trials should be used?

The number of trials of an experiment determines the accuracy of parameter estimation: The more data are entered into an analysis the more accurate all parameters can be estimated. In a recent set of simulation studies, Lerche et al. (submitted) found that for parsimonious models with few parameters reasonably accurate estimations were possible with only 48 trials or sometimes even less. In most situations, a good accuracy is reached with 200 trials.

The recommended trial number depends on several aspects of the present data (Lerche et al., submitted): Firstly, if data is contaminated by trials in which participants do not use a continuous information sampling (but, e.g., a guess), more data are required. This is even the case, when these contaminants are no outliers in a statistical sense. Imagine, for example, a participant that uses a diffusion-like information sampling strategy in 95% of all trials, but bases his responses on guesses in the remaining 5%. Because guesses involves other (and probably faster) cognitive processes, the RT distribution from the guess trials will differ from the RT distribution of the judgement trials. If, however, both distributions overlap it will not be possible so identify the guessing trials on basis of RTs.

A second determinant of the required trial number lies in the scientific question that is addressed: If estimates need to have a high reliability (e.g., because inter-individual differences are in the focus of a study) larger trial numbers might be necessary. Thirdly, if data are mapped as correct vs. incorrect (see above) the absence of error responses will make a precise estimation of parameters difficult. Therefore, enough trials should be used so that each participant makes several errors. Finally, one has to consider that some parameters are more difficult to estimate than others: While, for example, the duration of non-decision-times can be estimated with high accuracy from medium trial numbers (*n* ≈ 100), very large trial numbers (*n* > 1000) are often required to estimate the inter-trial-variability parameters of drift and starting point with satisfactory accuracy.

### Step 3: data pre-treatment

Results from diffusion model analyses can be biased strongly when data is contaminated ((Ratcliff and Tuerlinckx, 2002); Lerche et al., submitted). Especially fast outliers have a strong impact and should be removed. Because of the positive skew of RT distributions fast outliers might be missed with typical procedures (e.g., inspecting box plots or *z*-scores). Therefore, RTs should be log transformed before an outlier analyses (for the diffusion model analyses, of course, the untransformed data has to be used). Another possibility is to find a point at the lower edge of the RT distributions where performance rises above chance level (Ratcliff and Tuerlinckx, 2002).

A careful outlier analysis is of special importance when the parameter estimation is based on a maximum-likelihood procedure; on the contrary, the KS method proved to be very robust (Lerche et al., submitted).

### Step 4: defining your model: choosing free parameters

The degree of complexity of a model depends on several factors. On the one hand, a model should not oversimplify reality: When important parameters are neglected (i.e., fixed to a specific but wrong value), effects will be forced on other parameters and thus results become invalid. Imagine, for example, a situation where there is a decision bias but the relative starting point is fixed to *z*_*r*_ = 0.5 (indicating an absence of a decision bias). The decision bias would make responses at the preferred threshold faster; to account for this, the drift for “preferred” (“unwanted”) stimuli would be overestimated (underestimated). Thus, results from the restricted model would erroneously indicate a bias in terms of information processing.

On the other hand, models should be defined as parsimonious as possible, because many free parameters might lead to overfitting and make results unstable, especially if not enough trials are used. For example, model fit might be excellent no matter if you allow for a decision bias (i.e., asymmetric starting points) or for a perceptual bias (i.e., different drift for different stimulus types). In our experience, for small and medium trials numbers (<500) setting inter-trial-variability of drift (*s_*v*_*) and starting point (*s*_*zr*_) to zero makes the estimation of the remaining parameters more robust, even if there is an inter-trial-variability in data. Note that this is not the case for inter-trial-variability of non-decision time (*s*_*t*0_). Because *s*_*t*0_ has a great impact on the shape of the RT-distribution it is often harmful to neglect this parameter. Additionally, the difference in non-decision time for upper and lower threshold (*d*) can usually not be estimated simultaneously with starting point (Voss et al., 2010); therefore you should set either *d* = 0 or *z*_*r*_ = 0.5, whatever seems theoretically more plausible (large trial numbers might allow to estimate both parameters simultaneously).

Decisions of model complexity get more complicated when different types of stimuli or different experimental manipulations are compared. In this case, the researcher has to decide which parameters are allowed to vary between conditions. If, for example, an experiment comprises “easy” and “difficult” trials, it is plausible that this affects the drift, and different drift parameters should be estimated for different trial types. However, decisions on this matter need careful consideration, because false fixations will again lead to invalid results. Whenever it is the aim of a study to check on which parameters a manipulation maps, we recommend to model data from the different conditions completely independently (allowing for all parameters to vary between conditions). A disadvantage of estimating completely independent models for all conditions is than not all available information is used, and power to find relevant differences might be reduced. A discussion of this problem is given by Donkin et al. (2011, 2014).

### Step 5: choosing an optimization criterion

On this step, the researcher needs to decide which software or algorithm to use for the parameter estimation. This decision may depend on the number of trials and the quality of data (Lerche et al., submitted). For large data sets (>500), always robust procedures (like KS or CS) are recommended. For small data sets (<100) chi-square based approaches will not work properly, and maximum likelihood procedures may be good option if one is confident that data are not contaminated, and the Kolmogorov-Smirnov approach should be used, when a more robust procedure is required.

### Step 6: assessing model fit

In diffusion model application, the assessment of model fit should be a mandatory step. It is problematic to use the standard statistical tests associated with the chi square or Kolmogorov Smirnov criteria here, because results strongly depend on numbers of trials: For small data sets the power is too small to reliably detect misfit, and for large datasets deviations will nearly always be significant. Therefore, either graphical inspection or Monte Carlo simulations provide better alternatives.

Graphical inspection can be done for each individual by so-called quantile-probability plots (e.g., Ratcliff and Smith, 2010). These graphs show different quantiles of the empirical and predicted RT distributions as a function of the probability of correct (or erroneous) responses (for different stimulus types). If an experiment comprises data from many participants, we recommend using scatter plots that plot predicted values against empirical values for the 25, 50, and 75 quantiles of the RT distributions and for accuracy of responses (e.g.,Voss et al., 2013b, Appendix B). When all data points are positioned near the main diagonal, a good fit can be assumed.

The assessment of model fit with Monto Carlo simulations has the advantage that it leads to a clear criterion for which participants there is a satisfactory model fit. To this end, a critical value for an acceptable fit has to be determined. This critical value will depend on the number of trials, conditions, and parameters, on the estimation procedure, and possibly as well on the observed range of parameter values. Therefore, datasets have to be simulated that match the empirical data sets as closely as possible. It is recommended to draw at least 1000 parameter sets from a multidimensional normal distribution defined by the covariance matrix of the estimated parameter values. This can be accomplished, for example, by the *mvtnorm* library from the *R* environment. Then, for each of the 1000 parameter-sets one data set is simulated. The *construct-sample* tool of *fast-dm* can be used for this purpose (see below; note that each condition must be simulated separately and combined later into one file). In the next step, simulated parameter sets are entered into a diffusion model analysis with the same settings as used for the analysis of empirical data. From the results, only the fit indices are of importance: The 5% quantile of the distribution of fit indices is then used as critical value to assess fit of empirical results: All data-sets performing worse than this 5% criterion should be regarded as bad fitting. If notably more than 5% of data sets show bad fit, it should be questioned critically whether the diffusion model is suitable for the task.

### Step 7: interpretation of results

The last step of the diffusion model analysis is the interpretation of results. Typically, parameters are estimated for each individual; in this case estimates can be entered as dependent measures into statistical analyses (e.g., ANOVA) to check for differences between conditions. Alternatively, it is possible to compare model fit (e.g., BIC) between models with different restrictions to see which restrictions lead to a notable decrease of model fit.

## Using *fast-dm-30*: a user's manual

### Overview

When *fast-dm* is started, it reads commands from an external *control file* (named by default *experiment.ctl*). Commands in the *control file* control program settings, specify parameters that are estimated or fixed to given values, and set file names for input and output. *Fast-dm* can be started by double clicking on the program icon; in this case, the control file *experiment.ctl* will be read from the directory in which *fast-dm* is started. If no such file exists, *fast-dm* terminates immediately. Generally, we recommend starting *fast-dm* from a command console.^3^ Otherwise, error or warning messages can be lost because these are presented only on the screen in a window that closes as soon as *fast-dm* terminates. From a command window, the program is started by typing “fast-dm” (within the correct directory). You can add the file name of a control file as command line option: For example, “fast-dm exp1.ctl” will start *fast-dm* with the control file *exp1.ctl*.

Generally, the following steps are necessary to use *fast-dm*.

1. Create a directory for your analyses.
2. Save all data files and a copy of *fast-dm* in this directory.
3. Create a control file with a text editor (see below).
4. Start *fast-dm* (optimally from a command window).
5. Read results into your favorite statistics software for further analysis.

### License, source code, and compiled binaries

*Fast-dm* is free software; you can use, redistribute it and or modify it under the terms of the GNU General Public License. Details are given in the file *COPYING* that is included in the download archives. In the Downloads section of the *fast-dm* homepage (http://www.psychologie.uni-heidelberg.de/ae/meth/fast-dm/index.html) we provide three different zip-files. The first, labeled as “Windows Binaries,” contains the precompiled executable files for Microsoft Windows systems. Specifically, we provide the programs *fast-dm.exe* (for parameter estimation), *construct-samples.exe* (for the simulation of data samples), *plot-cdf.exe* (for generating a cumulative distribution function from a set of parameter values), and *plot-density.exe* (for the generation of the density function from a set of parameter values). You may need to install the Microsoft Visual C++ Redistributable package for Visual Studio 2012 (http://www.microsoft.com/en-us/download/details.aspx?id=30679) to get these programs running.

Secondly, we provide the complete C source code of *fast-dm* in the “source” archive. Together with the source code files, this archive contains short instructions (file INSTALL) on compiling *fast-dm* on Unix-like systems (e.g., Linux and MacOS), and a short manual (file MANUAL).

Finally, we provide a Visual Studio 2012 Project (including source code files and reasonable project setting) for Windows users who want to modify the software. To make use of this, Microsoft Visual Studio 2012 needs to be installed, which is freely available in the Express edition (http://www.microsoft.com/en-us/download/details.aspx?id=34673).

### Data files

**Figure 1:**
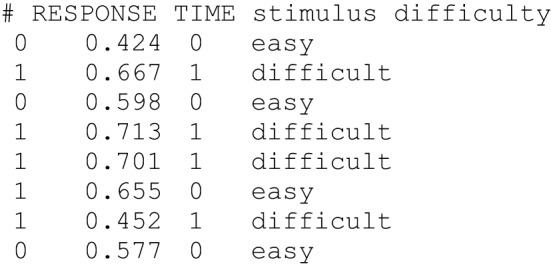
**Example of the first lines of a data file**. Lines starting with “#” are ignored. The RESPONSE (0 = “lower threshold,” 1 = “upper threshold”) and TIME column (response time in seconds) are mandatory. Further columns can be added to give information about the stimulus (e.g., 0 = “word” vs. 1 = “non-word”) or experimental condition (e.g., “easy” vs. “difficult”).

Data is read from plain text files. Each line of a *data file* contains information from one trial, and data columns have to be separated by blanks or tabs (see Figure 1 for an example of a *data file*). Lines starting with a hash mark (#) are considered as comments and are ignored. Each data file needs to comprise at least two columns: One column—referred to as “RESPONSE” column in the *control file*—contains information about responses coded as 0 and 1 for the lower and upper threshold, respectively. The second required column—labelled as “TIME” column in the control file—gives response times in seconds. Optionally, further columns can be added containing information about stimulus types (e.g., “word” vs. “non-word”) and/or the experimental conditions (e.g., “speed instruction” vs. “accuracy instruction”). In these additional columns either words or numbers can be used for coding different conditions.

*Fast-dm* estimates parameters independently for separate *data files*. Usually, each *data file* will contain data from one participant. However, sometimes it may be a good idea to split data from one participant into separate files, so that independent models are estimated for different conditions.

### Control files

**Figure 2:**
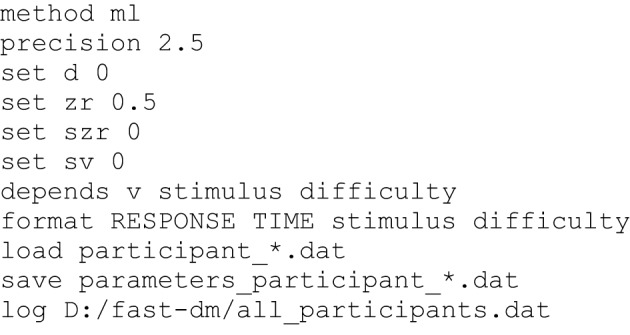
**Example of a control file**. The maximum likelihood criterion is used with (reduced) precision 2.5. Four parameters (*d*, *z*_*r*_, *s*_*zr*_, *s*_*v*_) are fixed to given values. Drift is free to differ depending on *stimulus* and *difficulty*. If both conditions have two values (see Figure 1), 2 × 2 = 4 different values for the drift will be estimated, whereas for the remaining parameters (*a*, *t*_0_, *s*_*t*0_) one value is estimated for all conditions (resulting in seven free parameters). The remaining commands specify the format of data files, and file names for data, save, and log files.

To run *fast-dm*, a *control file* is required containing commands that specify settings for the parameter estimation process. This *control file* is a plain text file that can be constructed with any text editor (see Figure 2 for an example of a *control file*). Each line of a control file contains a *fast-dm* command and additional values specifying the chosen settings (separated by blanks). As in *data files*, lines starting with a hash mark (#) are ignored. Table 2 gives an overview of all commands with explanations and examples. Some commands are required (*format*, *load*, and *save* or *log*), while others are optional. In the command file, the definition of the model (*depends* and *set* commands) have to precede the *format* command, and *load* and *save/log* commands must come after. All other commands can be placed anywhere in the *control file*.

**Table 2:** **Commands of the control file**.

**Command**	**Description**	**Examples**
method *CRITERION*	Determines the optimization criterion (ml, Maximum Likelihood; ks, Kolmogorov-Smirnov; cs, Chi-Square); default setting: method = ks	• method ml
		• method cs
precision *VALUE*	Defines the precision of the calculation; default setting: precision = 3	• precision 2.5
		• precision 5
set *PARAMETER VALUE*	Fixes a parameter to a specific value; default setting: no fixations	• set d 0
		• set zr 0.5
		• set szr 0
depends *PARAMETER CONDITION*	Denotes that a parameter may vary between different conditions; default setting: parameters do not depend on conditions	• depends *t*_0_ block
		• depends *v* stimulus difficulty
format *CONDITION*…	Defines columns of the data file(s). The command requires the variables RESPONSE and TIME	• format RESPONSE TIME
		• format RESPONSE TIME stimulus difficulty
		• format * RESPONSE TIME
**load** *FILE_NAME*	Declares the names of the input files	• load participant_1.dat
		• load participant_*.dat
**save** *FILE_NAME*	Defines the names of separate output files (one output file for each data set)	• save parameters_participant_1.dat
		• save parameters_participant_*.dat
**log** *FILE_NAME*	Defines the name of a common output file (one output file for all data sets)	• log all_participants.dat

Commands in bold font are required while the other commands are optional.

The *method* command specifies the optimization criterion. Possible values are “ml” for Maximum Likelihood, “ks” for Kolmogorov-Smirnov, and “cs” for Chi-Square. Depending on the chosen method, the appropriate criterion is given in the output. If no method is specified, KS is chosen be default.

The *precision* command controls the accuracy of calculation of predicted CDFs (for the KS and CS method) or DFs (for the ML method). Any positive real numbers can be used as arguments, with higher precision values leading to a higher accuracy and longer duration of calculation. Reasonable values range from about 2.0 to 5.0. We tuned the calculation routines to achieve an error in calculated values that is approximately ε = 10^−precision^ (however, we cannot guarantee that this bound is always strictly observed). The command is optional; if no precision is specified, a default value of *precision* = 3 is used.

With the *set* command, parameters are fixed to given values. The command requires two arguments (separated by blanks): First, the name of the parameter is given (see Table 1 for the *fast-dm* notation for all parameters), followed by the desired value. For example, “*set zr 0.5*” fixes the relative starting point to 0.5, that is, the process starts at 0.5 · a and is thus assumed to be unbiased. Parameters that are fixed to a value are not estimated by *fast-dm*. Generally, we recommend fixing either *d* to 0 or *z_*r*_* to 0.5 because it is difficult to estimate both parameters simultaneously (Voss et al., 2010). In case of small trial numbers, it often makes sense to make a model as parsimonious as possible. For this purpose it might help to additionally fix *s_*z*_* and *s_*v*_* to 0 because these parameters have only minor impact on the predicted distributions and can only be reliably estimated from huge data sets (Voss et al., 2013a). The *set* command is optional; by default all parameters are estimated. The *set* command can be used repeatedly to fix different parameters.

With the *depends* command parameters can be specified that are estimated separately for different types of stimuli or different experimental conditions. The *depends* command must be followed by a parameter name and by user-chosen labels for the conditions. Parameters can depend on different factors (e.g., type of stimulus and block of the experiment); in this case, labels for each factor are specified one after another (separated by blanks). For each parameter that can vary between conditions, a separate *depends* command must be specified. All condition labels that are used in any *depends* command must be specified as a column in the data file(s) with the *format* command (see below). The *depends* command is optional. By default, all parameters are assumed to be equal across all experimental conditions.

The *format* command defines the columns of the data file(s). The labels RESPONSE and TIME are mandatory (capital letters are required for these). Additionally, all factor labels used in *depends* commands have to be named here as well (capitalization must be identical in the *format* command and the *depends* commands). Columns that shall be ignored by *fast-dm* can be assigned with any new name or with an asterisk (^*^). The *format* command is required and needs to be placed after all *set* and *depends* commands but before *load, save*, and *log*.

The *load* command specifies the file name(s) of data files. *Fast-dm* tries to load data from the directory in which it is started, unless a path is given. File names may contain asterisks (e.g., “participant_*.dat”); in this case, the asterisk is a wildcard character that can be replaced by any number of characters. Any matching files within the chosen directory will be loaded. The *load* command is required.

To save results, the *save* or the *log* command (or both) have to be used. With the *save* command, separate output files are generated for each data file. When the data file name as specified in the *load* command contains an asterisk, an asterisk is also required in file name defined in the *save* command, so that multiple file names for output can be generated. With the *log* command, one common output file is generated that contains estimated parameter values as a table that can be read from any statistical software for further analyses of results.

### Output

**Figure 3:**
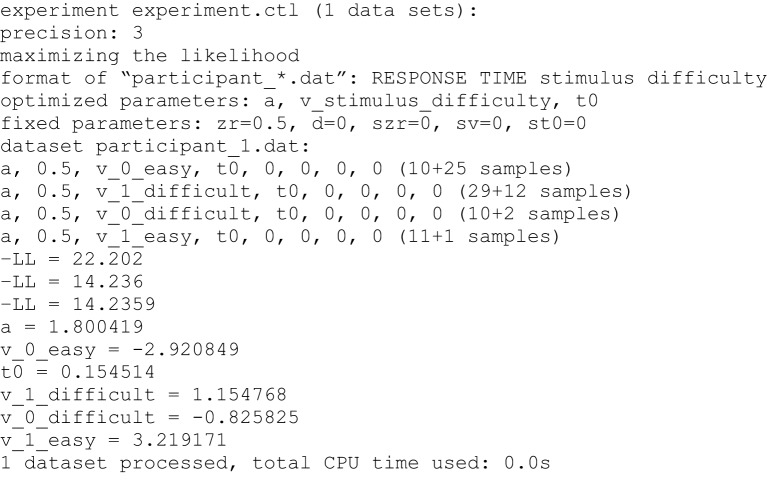
**Example of the console output**. First, information on the selected control file, the precision and method of estimation, the format of the data files and the estimated and fixed parameters are given. Any parameter depending on a condition is indexed with the name of the condition variable(s). Estimated parameters and numbers of responses at lower and upper threshold are presented separately for each condition. The three “-LL” values result from the three consecutive runs of the simplex algorithm. In the following lines the estimated values for all parameters are displayed. Finally, the number of processed data sets and the required computational time is given.

The output of the estimation procedure is shown directly in the console (Figure 3). First, the name of the *control file* and central characteristics of the estimation procedure are presented (precision, method of estimation, format of data files, estimated and fixed parameters). Then, parameters that are estimated within each condition of an experiment are listed (numbers represent fixed parameters). For parameters that depend on conditions the labels of conditions as found in the appropriate columns of the data files are attached to the parameter identifier. At the end of these lines, the number of observed responses at lower and upper threshold (coded with 0 or 1 in the data file, respectively) within each condition are presented.

Following the model specifications, fit values resulting from each of the three consecutive runs of the parameter search are displayed. If the KS criterion has been selected, the (combined) *p*-values of the KS distances will be presented. We warn not to take these *p*-values as a direct indicator of significant model misfit (Voss et al., 2013a): Firstly, if multiple conditions are used the presented *p*-value is the product of *p*-values from all conditions, which may lead to very small combined values, even if the single KS statistics from all conditions are not significant (e.g., *p* = 0.10 · 0.35 · 0.12 · 0.60 = 0.002). On the other hand, *p*-values would be too liberal, if—as is done here—the forms of predicted functions are fit to the empirical functions before the KS statistic is determined, which may possibly prevent statistical significance. If the ML method is chosen the presented fit index is -LL. Small values indicate a good fit. Finally, when selecting CS as optimization criterion the chi-square values will be displayed; here smaller values again indicate better fitting. If no valid model is found (e.g., if the likelihood for at least one RT is zero), a penalty value is presented instead of the fitting index.

After the third run of the parameter search is finished the resulting estimates for all parameters are shown. If multiple data sets are processed, the estimates will be presented one after the other. Finally, the total computation time is presented.

If the user wrongly defines a command (e.g., a condition is named in the *depends* command which has not been assigned to a data column in the *format* command) an error message appears and the program is aborted. Furthermore, a warning message will be presented and the estimation process stopped if the number of trials is not sufficient for parameter estimation. For the ML and KS methods, for each experimental condition at least 10 trials are required (no matter whether responses vary between trials or not). For the estimation with CS as optimization criterion at least 12 trials sharing the same response are required (i.e., 12 trials with all 12 responses at the upper threshold would be ok, while 20 trials with 10 responses at each threshold cannot be analyzed using the CS method).

**Figure 4:**
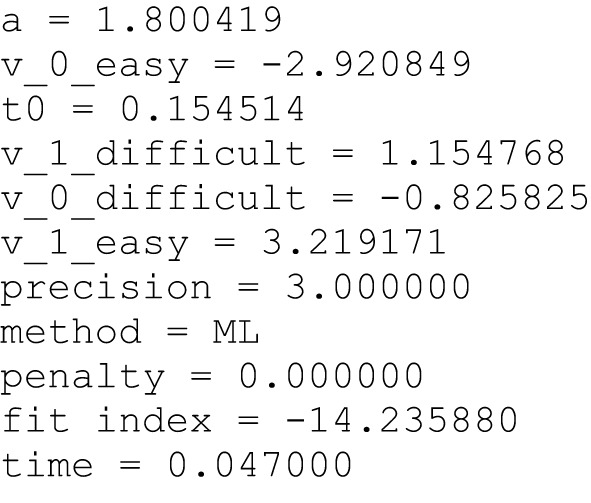
**Example of a save file**. When the save command is used for each data file a separate output file is generated containing a short version of the screen output (see Figure 3).

**Figure 5:**
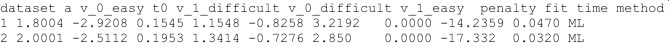
**Example of the beginning of a log file**. When the log command is used one common file containing the estimates from all data files is generated. This is especially convenient for further statistical analyses.

Besides the output on the screen the results are also saved in files, either separately for each data file (using the *save* command in the control file; see Figure 4) and/or in one summary file including the estimates of all data files (using the *log* command; see Figure 5).

### Additional tools

*Construct-samples, plot-cdf, and plot-density* are command-line tools which can be downloaded from the “*fast-dm* Downloads” section (archive “Windows binaries”; source code is also available in the “source” archive). The programs need to be started from the command console, and all settings are entered directly as command line arguments.

#### Making simulations with construct-samples

*Construct-samples* allows simulating data sets for a given parameter set. This is useful (1) to evaluate the quality of parameter recovery of *fast-dm* and (2) to get a distribution of fit-values that allows assessing the fit of models estimated from empirical data. For these purposes data sets have to be simulated from known parameter values. Then, *fast-dm* is applied to the simulated data sets and the estimated parameter values are compared to the true values from the simulation.

If this tool is started by just typing *construct-samples* into the command line, all default settings are used (see Table 3). Typically, however multiple command line options will be entered at starting *construct-samples*. Options start with a minus sign, followed by a letter and in most cases by an additional argument, typically a number (exceptions: -r has no additional argument and -o needs a string determining the file name).

**Table 3:** **Command-Line Options for construct-samples, plot-cdf and plot-density**.

**Option**	**Description**	**Default**
-a *VALUE*	*VALUE* is assigned to parameter *a*	1
-z *VALUE*	*VALUE* is assigned to parameter *z_*r*_*	0.5
-v *VALUE*	*VALUE* is assigned to parameter *v*	0
-t *VALUE*	*VALUE* is assigned to parameter *t*_0_	0.3
-d *VALUE*	*VALUE* is assigned to parameter *d*	0
-Z *VALUE*	*VALUE* is assigned to parameter *s_*zr*_*	0
-V *VALUE*	*VALUE* is assigned to parameter *s_*v*_*	0
-T *VALUE*	*VALUE* is assigned to parameter *s_*t*_*_0_	0
-p *VALUE*	The computational precision is set to *VALUE*	4
-n *VALUE* ^a^	*VALUE* defines the trial number per data set	100
-r^a^	A random data set is generated	A deterministic data set is generated
-N *VALUE*^a^	*VALUE* defines the number of random data sets	1
-o *FILE_NAME*	The generated data is not presented in the console but saved to *FILE_NAME*	The generated data is presented in the console window but not saved

a Expression cannot be applied for plot-cdf and plot-density.

Command-line options are used to set parameter values for the simulation. Please note that notation differs here slightly from the usual *fast-dm* labels. This is because only one-letter commands can be used here. Therefore, “-z” is used for *z_*r*_*, “-t” for *t*_0_, and capital letters “-Z,” “-V,” and “-T,” for the intertrial variabilities *s_*zr*_*, *s_*v*_*, and *s_*t*_*_0_, respectively. The “-r” argument ensures that random samples are generated. This is what normally is needed for simulations. If “-r” is not present, a deterministic data set is calculated, where response times reflect directly the quantiles of the predicted distributions. With “-p” the precision of calculation can be adapted as in *fast-dm*. The number of trials within each simulated data set is set by “-n,” and the number of data sets is defined by “-N.” The file name(s) for output are determined with the “-o” command. If multiple data sets are generated, it is necessary to include “%d” in the name, which is then replaced by a different number for each data set (from 0 to *N*-1). If “-o” is not used, results are presented in the console only. Results always comprise two columns: The first is coding simulated responses (0 vs. 1) and the second gives the response times in seconds. Finally, a short help page can be opened by typing “construct-samples -h.”

For example, *construct-samples* could be started by typing the following command:

construct-samples -a 2 -z 0.5 -v 3 -t 0.5 -r -n 250 -N 1000 -o %d.sim

With this command, 1000 data sets named *0.sim* to *999.sim* are generated containing random samples of 250 trials simulated from parameter values *a* = 2, *z_*r*_* = 0.5, *v* = 3, and *t*_0_ = 0.5 (for *d* and intertrial variabilities the default values of 0 are assumed).

Often, you will need to simulate data sets for more complex situations. Imagine, for example, that multiple conditions with different parameter values should be simulated. To do so, you need to simulate data separately for each condition and then combine data sets into common files. This can be done automatically—for example—using *R*. The application of *construct-samples* (and *fast-dm*) from the *R* environment is illustrated in the examples that can be downloaded from the *fast-dm* website.

### Plotting (combined) CDFs with *plot-cdf and plotting DFs with plot-density*

*Plot-cdf* can be used to calculate values of predicted CDFs of a certain parameter set. This can be useful to demonstrate model fit graphically: If predicted and empirical CDFs are plotted in the same diagram, it is possible to assess whether both curves match sufficiently well, and—if not—where the main differences are (see Voss et al., 2008, for an example of this strategy). Note that *plot-cdf* generates so-called combined CDFs, where distributions from lower and upper threshold are merged by multiplying all RTs from the lower threshold by -1 (Voss et al., 2004; Voss and Voss, 2007).

Command line options are very similar to those of *construct-samples* (see Table 3). The only differences are that -r, -n, and -N cannot be used with *plot-cdf*. For example,

plot-cdf -a 2 -z 0.5 -v 3 -t 0.5 -o cdf.dat

generates values for a predicted CDF with *a* = 2, *z*_*r*_ = 0.5, *v* = 3, and *t*_0_ = 0.5 and saves these values into a file named “cdf.dat.” Output consists of two columns: The first contains the reaction times (with negative values indicating responses at the lower threshold). The second column displays the cumulative probability values. For graphic diagrams output from *plot-cdf* has to be entered in other programs like *R* or *Excel*.

The *plot-density* tool can be used to get values for the density functions at upper and lower threshold. Command line options are identical to those in *plot-cdf*. Therefore,

plot-density -a 2 -z 0.5 -v 3 -t 0.5 -o density.dat

will save the density functions for the same settings as used in the CDF example. Here, the output comprises three columns that contain values for predicted response times and density functions at upper and lower threshold (densities at the lower threshold get a negative sign here).

## Concluding remarks

After 6 years of using *fast-dm*, several optimizations have been made improving the performance and functionality of the program. The most important extension is the inclusion of different optimization criteria (Maximum Likelihood, Kolmogorov-Smirnov, and Chi-Square). This can potentially improve results from diffusion model analyses greatly, because all criteria have different advantages and shortcomings, and now the criterion that is best for a given data set can be chosen. Obviously, the number of trials is an important factor for this choice. Often, ML will outperform the other methods at small data sets. Secondly, purity of data will influence quality of results as well: When RTs are contaminated, ML can be strongly biased (Ratcliff and Tuerlinckx, 2002), while both other methods will probably be more robust. Further factors, like the number of estimated parameters, the number of experimental conditions, the task difficulty (i.e., percentage of errors) will also influence the accuracy of parameter recovery. However, it is less clear how these factors influence performance of the different criteria. Future simulation studies are essential to allow an informed choice of the best criterion.

In the development of *fast-dm* we did not (yet) program a graphical user interface. We are aware that this might be seen by some as a barrier to the application of the program. The main reason for us to develop *fast-dm* without graphical user interface was to ensure that the program can be compiled within any operating system. We hope that many users of *fast-dm* find it usable and helpful and that *fast-dm* thus helps to promote diffusion model analyses as a powerful method to infer cognitive processes.

### Conflict of interest statement

The authors declare that the research was conducted in the absence of any commercial or financial relationships that could be construed as a potential conflict of interest.

## Footnotes

^1^Strictly speaking, it is also possible to use a different parameter (*a* or *v*) to define the scale; in this case, intra-trial variability of the drift (*s*) can be estimated as a free parameter (Donkin et al., 2009b).

^2^Strictly speaking, this is not an exact implementation of a chi-square criterion because bins are defined by the data (and not by predicted distributions). However, the resulting values approximate nonetheless a chi-square distribution, and parameter estimates do not differ substantially (Ratcliff and Childers, in press), while computation is much faster.

^3^(Ratcliff and Childers, in press). Windows users can open a command window by typing “cmd” in the start menu.
